# Traditional Chinese Manual Therapy (Tuina) reshape the function of default mode network in patients with lumbar disc herniation

**DOI:** 10.3389/fnins.2023.1125677

**Published:** 2023-03-15

**Authors:** Xiao-Min Chen, Ya Wen, Shao Chen, Xin Jin, Chen Liu, Wei Wang, Ning Kong, Dong-Ya Ling, Qin Huang, Jin-Er Chai, Xiao-Lei Zhao, Jie Li, Mao-Sheng Xu, Zhong Jiang, Hong-Gen Du

**Affiliations:** ^1^Department of Tuina, The First Affiliated Hospital of Zhejiang Chinese Medical University (Zhejiang Provincial Hospital of Chinese Medicine), Hangzhou, China; ^2^Department of Radiology, The First Affiliated Hospital of Zhejiang Chinese Medical University (Zhejiang Provincial Hospital of Chinese Medicine), Hangzhou, China; ^3^Department of Radiology, Changshu No.2 People’s Hospital, The Affiliated Changshu Hospital of Xuzhou Medical University, Changshu, Jiangsu, China

**Keywords:** lumbar disc herniation, resting-state fMRI, regional homogeneity, functional connectivity, Tuina

## Abstract

**Purpose:**

Investigating the changes of regional homogeneity (ReHo) values and both static and dynamic functional connectivity (FC) before and after Traditional Chinese Manual Therapy (Tuina) in patients with lumbar disk herniation (LDH) through resting-state functional magnetic resonance imaging (RS-fMRI). Based on this, we observe the effect of Tuina on the above abnormal changes.

**Methods:**

Patients with LDH (*n* = 27) and healthy controls (HCs) (*n* = 28) were recruited. The functional magnetic resonance imaging (fMRI) scanning was performed two times in LDH patients, before Tuina (time point 1, LDH-pre) and after the sixth Tuina (time point 2, LDH-pos). And for one time in HCs which received no intervention. The ReHo values were compared between LDH-pre and HCs. The significant clusters detected by ReHo analysis were selected as seeds to calculate static functional connectivity (sFC). We also applied the sliding-window to perform dynamic functional connectivity (dFC). To evaluate the Tuina effect, the mean ReHo and FC values (both static and dynamic) were extracted from significant clusters and compared between LDH and HCs.

**Results:**

In comparison to HCs, LDH patients displayed decreased ReHo in the left orbital part middle frontal gyrus (LO-MFG). For sFC analysis, no significant difference was found. However, we found decreased dFC variance between LO-MFG and the left Fusiform, and increased dFC variance in the left orbital inferior frontal gyrus and left precuneus. Both ReHo and dFC values revealed after Tuina, the brain activities in LDH patients were similar to HCs.

**Conclusion:**

The present study characterized the altered patterns of regional homogeneity in spontaneous brain activity and those of functional connectivity in patients with LDH. Tuina can reshape the function of the default mode network (DMN) in LDH patients, which may contribute to the analgesic effect of Tuina in LDH patients.

## Introduction

Lumbar disc herniation (LDH), defined as the displacement of lumbar disc material beyond the limits of the disc space, is one of the most common spinal disorders and significantly affects the quality of life of patients and the social security system (Al Qaraghli and De Jesus, 2022). Surgery or conservative treatment is used to treat this disease. Most patients with LDH prefer conservative treatment because it is non-invasive and effective (Gadjradj et al., 2017; Wan et al., 2022).

One of the most commonly used conservative treatment methods is Traditional Chinese Manual Therapy (Tuina). The performer rolls, pinches, and squeezes the skin and paravertebral muscles of the patient’s lumbar vertebrae to relax the muscles and intervertebral joints around the lumbar vertebrae and pulls diagonally to correct dysfunctional joints and promote recovery of protruding discs. A systematic review found that spinal massage can significantly improve pain and dyskinesia in patients with low back pain, making it an indispensable method in non-surgical therapy (Paige et al., 2017; Rubinstein et al., 2019; de Zoete et al., 2021). Studies have proven that Tuina improves biomechanics (correction of bone dislocation, relief of nerve root compression caused by protrusions, relief of muscle contracture of paravertebral tissues) and promotes the absorption of endogenous inflammatory substances (Rong et al., 2017; Ke et al., 2021).

In recent years, the application of resting-state functional magnetic resonance imaging (RS-fMRI) has made it possible to study neural activity in a living brain without causing injury. According to studies, patients with LDH have abnormal brain activity. Zhou et al. found that patients with LDH had increased Amplitude of Low-Frequency Fluctuations (ALFF) abnormalities in the pain matrix and information processing areas and decreased ALFF in the default mode network (DMN) (Zhou et al., 2018). According to Zhang et al., LDH-related chronic sciatic syndromes can cause regional brain changes involving self-referential, emotional responses, and pain regulation functions (Zhang et al., 2022). However, there is still no unified conclusion on the changes in brain activity caused by LDH. On the other hand, existing studies mainly focus on changes in brain function in the LDH disease state and do not examine whether normal brain activity can be restored after effective treatment. Therefore, further elucidation of the characteristics of brain activity changes under LDH status and verification of the reversibility of abnormal brain activity changes have important guiding importance for clinical practice.

Our previous research has shown that the lingual gyri and left cerebellum of LDH patients have abnormally high Crus1 ALFF and fractional ALFF (fALFF) in the slow-4 and conventional bands. By comparing the changes in abnormal brain activity before and after spinal manipulation, the increase of ALFF and fALFF in abnormal brain activity decreased to normal levels after Tuina (Wen et al., 2022). In this study, we focused on the intensity of spontaneous activity in local brain regions but paid no attention to the coherence of spontaneous activity in local brain regions or the correlation between brain activities in different areas.

An indicator of spontaneous brain activity is called regional homogeneity (ReHo), which assesses the degree of local synchronization between the closest neighboring voxels (e.g., 7,9,27 voxels). The modification of the temporal coherence of the local neuronal activity is indicated by an increase or decrease in the ReHo value (Zang et al., 2004). ReHo has been shown to be highly reproducible, sensitive, and reliable for identifying local functional activities (Zuo and Xing, 2014; Jiang and Zuo, 2016). Researchers found that abnormal ReHo in specific brain regions may be related to pain processing in patients with this condition (Zhang S. S. et al., 2014) and that impaired ReHo was related to pain severity (Zhou et al., 2019). ReHo can successfully reveal pain-related pathological mechanisms, according to evidence from these studies. To determine the functional connections between brain regions, functional connectivity (FC) calculates the correlation coefficient of the blood oxygen level-dependent (BOLD) time series between a specific starting point and all other voxels or brain regions of interest (ROIs) (Shahhosseini and Miranda, 2022). Growing evidence suggests that the brain is a dynamic system that quickly switches discrete models (Hutchison et al., 2013; Vidaurre et al., 2017). Thus dynamic indices based on sliding-window shifts can better reflect the brain’s information processing efficiency and is more insightful than the static index (Lindquist et al., 2014). Our understanding of the intricate process by which spinal massage controls brain activity may be enhanced by RS-fMRI research on static functional connectivity (sFC) and dynamic functional connectivity (dFC).

In this study, we first identified the different brain regions by calculating Reho and used the different brain regions as seeds to study the static and dynamic functional connectivity between different groups. Our research may reveal whether the brain activity changes of LDH patients are reversible, and whether effective Tuina can correct the brain activity of LDH disorders.

## Materials and methods

### Participants

In our study, 30 LDH patients and 30 healthy controls (HCs) were recruited from the First Clinical Medical College of Zhejiang Traditional Chinese Medical University from 24th November 2020, to 17th August, 2021. Both groups were matched with age, years of education and gender. All the patients were required to complete two clinical assessments, Visual Analogue Scale (VAS) (Hawker et al., 2011), and the Chinese Short Form Oswestry Disability Index Questionnaire (C-SFODI) (Smeets et al., 2011), to evaluate the degree of pain and daily functional activities of participants.

The criteria for inclusion of patients with LDH were as follows: (1) right-handed; (2) aged between 20 to 60 years; (3) evidence of compression of the spinal canal on a lumbar MRI; (4) radiating pain from the lumbar region to the buttocks and lower limb; (5) positive in straight leg-raising test and augmentation test; knee and Achilles jerk reflexes weakened or missing; (6) VAS score ≥ 3/10; (7) C-SFODI score ≥ 20%; (8) not taking pain therapy for at least 1 month before the enrollment.The criteria for inclusion of HCs were as follows: (1) right-handed; (2) aged between 20 to 60 years; (3) no history of LDH; (4) no history of pain caused by any disease and haven’t accepted any pain-related treatment at least 1 month before enrollment.

The criteria for exclusion of all participants were as follows: (1) a history of spinal surgery or severe spinal trauma; (2) bone tuberculosis, tumor, severe osteoporosis, and other orthopaedics diseases; (3) combined with serious medical or psychiatric diseases, such as cardiovascular and cerebrovascular diseases, and those occur in the blood system and digestive system; (4) pregnancy or breastfeeding; (5) having autoimmune diseases, allergic diseases, acute and chronic infectious diseases; (6) having contraindications to functional magnetic resonance imaging (fMRI), such as metal implants, claustrophobia or devices in the body; (7) fMRI examination showing free nucleus pulposus and cauda equina syndrome; (8) vision loss and vestibular dysfunction;(9) acute or chronic pain caused by other diseases.

This study was approved by the Medical Research Ethics Committee and the Institutional Review Board of the First Clinical Medical College of the Zhejiang Traditional Chinese Medical University (No.2017-k-237-01) and was registered in Clinical Trial Registry (No. NCT03475095). We obtained written informed consents from all participants, and the study was conducted in accordance with the principles of the Declaration of Helsinki.

### Tuina interventions

**FIGURE 1:**
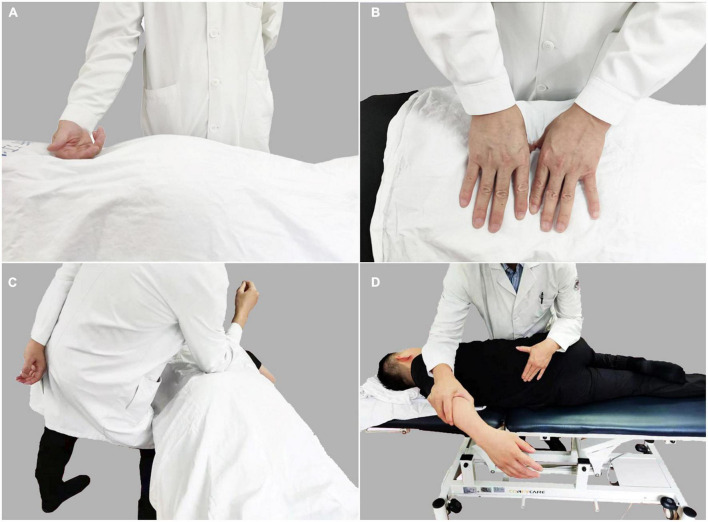
Tuina performed by rolling **(A)**, kneading **(B)**, pushing **(C)**, and the pulling and rotating **(D)** to relax the muscles in the lower back area for the purpose of alleviating pain and improving lumbar function.

We performed Tuina interventions using traditional Chinese Tuina which includes multiple manipulations (Lee et al., 2017). In the present study, the Tuina interventions were conducted to relax the lumbar muscles by rolling (Figure 1A), kneading (Figure 1B), pushing (Figure 1C) and the pulling and rotating manipulations (Figure 1D) were utilized to correct disordered spinal joints, to relieve pain and to improve lumbar function. Each Tuina session lasted approximately 25 min. All patients received a total of six Tuina sessions, and three times of Tuina proceeded in one week.

### Quality control of Tuina

Requirements for physician: Tuina was operated by the same physician who has more than 5 years of massage clinical practice experience. Moreover, the physician has received the standardized requirements of this specialty massage training and holds a physician’s certificate. Before conducting treatment for patients with LDH, the physician had to standardize the specific Tuina operation according to the operation essentials to maintain stability in strength, shape and frequency.

Requirements for Tuina environment and equipment: Multi-functional massage beds were used, along with proper adjustment of angle and height which were set to keep patients comfortable. Meanwhile, standard treatment towels and disposable bed sheets provided by Zhejiang Provincial Hospital of Chinese Medicine were used. In our study, all patients enrolled completed six Tuina sessions, and had no complications from Tuina sessions.

### MRI data acquisition

All participants underwent a 3T Siemens scanner (Verio, Siemens AG, Erlangen, Germany) with a 12-channel head coil. HCs group underwent one fMRI scan, while the LDH group needed to be scanned twice, before Tuina and after the sixth Tuina. The functional images were obtained using a gradient echo-planar imaging sequence. The scanning parameters were as follows: repetition time (TR) = 2,000 ms, echo time (TE) = 30 ms, 43 interleaved axial slices, matrix size = 64 × 64, field of view (FOV) = 220 mm × 220 mm, flip angle (FA) = 90°, slice thickness = 3.2 mm, gap = 0 (voxel size 3.4 × 3.4 × 3.2), number of volumes = 230. The structural images were obtained by a sagittal T1-weighted 3D sequence with magnetization prepared rapid gradient echo (MPRAGE). The scanning parameters were as follows: TR/TE = 8100/3.1 ms, sagittal slices, slice number = 176, matrix size = 256 × 256, FOV = 256 mm × 256mm, FA = 8°, slice thickness = 1 mm, gap = 0 (isotropic voxel size = 1 × 1 × 1). During rs-fMRI scanning, all participants were asked to keep their eyes closed, not to think about anything and not to fall asleep.

### Data preprocessing

The data preprocessing were performed using RESTplus V1.25 software (Jia et al., 2019) and included the following steps: (1) removing the first 10 volumes; (2) slice-time correction; (3) realignment; (4) normalization was performed using T1 image new segment; (5) removing the linear trend; (6) nuisance regression, including the white matter, the cerebrospinal fluid and Friston-24 head motion parameters (Friston et al., 1996); (7) filtering in the frequency range between 0.01 and 0.08 Hz; (8) spatial smoothing with a 6-mm full width at half maximum Gaussian smooth kernel (only for static and dynamic functional connectivity analysis).

### Regional homogeneity analysis

The Kendall’s coefficient of concordance (KCC) was used to measure the local synchronization of the time series of neighboring voxels as follows (Zang et al., 2004):


$$\text{W}=\frac{\sum {\left(R_{i}\right)}^{2}\;-\;n\,{\left(\bar{R}\right)}^{2}}{\frac{1}{12}\,K^{2}\,\left(n^{3}\;-\;n\right)}$$

where W is the KCC among given voxels, ranging from 0 to 1; *R_i_* is the sum rank of the *i*th time point; $\bar{R}$ (n+1) K)/2 is the mean of the *R_i_*; K is the number of time series within a measured cluster (K = 7, 19, and 27, respectively. K = 27 is used in the current study); n is the number of ranks. The ReHo value of each voxel was then divided by the global mean ReHo of each participant for standardization purposes. Noted that the spatial smoothing with a 6-mm isotropic full width at half maximum (FWHM) Gaussian kernel was performed after ReHo calculation.

### Static functional connectivity analysis

The significant clusters detected by ReHo analysis between LDH-pre patients and HCs were selected as seed regions of interest (ROIs) to calculate static functional connectivity. Briefly, for each participant, the mean time course of each ROI was correlated with the time courses of each voxel using Pearson correlation. The Fisher’s z-transform was applied to convert FC maps into Z maps to acquire optimum normality.

### Dynamic functional connectivity analysis

The significant clusters detected by ReHo analysis between LDH-pre patients and HCs were selected as seed regions of interest (ROIs) to calculate dynamic functional connectivity (dFC). The sliding window approach was applied to obtain the dFC maps for each participant by DynamicBC (V2.2)^1^ (Liao et al., 2014). We selected a window length of 50 TRs (100 s) and a window overlap of 98% (step size by 1 TRs) to compute the dFC of each participant (Li et al., 2018, Ma et al., 2021). The FC map for each participant were computed within each window, generating a series of FC maps. Subsequently, the coefficient of variance (CV) of dFC maps across time was calculated to measure the temporal variability of intrinsic brain activity. Finally, the dFC variability of all participants were then transformed into standardized z scores by subtracting the mean and dividing by the SD across each voxel to enhance data normality.

### Statistical analysis

The demographics and clinical variables were analyzed by the Statistical Package for the Social Sciences (SPSS) 22.0. The differences between the LDH patients and the HCs in age, years of education and clinical scores were tested with Student’s *t*-test. The gender difference was tested using the Pearson Chi-Square test.

For the ReHo analysis, we performed the two-sample *t*-tests to compare LDH-pre and HCs. Gaussian Random Field (GRF) was applied in multiple comparison corrections (voxel-level *p* < 0.05, cluster-level *p* < 0.05). The significant whole clusters obtained from ReHo analysis were defined as ROIs. For the static FC and dFC analysis, two-sample *t*-tests was also applied to compare LDH-pre patients and HCs. GRF was applied in multiple comparison corrections (voxel-level *p* < 0.05, cluster-level *p* < 0.05).

To evaluate the Tuina effect, the abnormal brain regions identified by group comparisons (LDH-pre vs. HCs) were created as brain masks separately. The mean ReHo and FC values (both static and dynamic) were extracted within the brain masks. Two-sample *t*-tests were performed to compare these values between LDH-pre and HCs, and those between LDH patients after Tuina interventions (LDH-pos) and HCs (*p* < 0.05). Paired *t*-test was used to compare mean ReHo values between LDH-pos and LDH-pre (*p* < 0.05). GraphPad Prism 8 was used to assess the changes of ReHo/FC values.

## Results

### Demographic and clinical characteristics

In total, 3 LDH patients and 2 HCs who had maximum head movement exceeding 3 mm or 3°were excluded from the subsequent statistical analysis. we ultimately included 27 LDH patients (17 males, age: 32.2 ± 9.5) and 28 HCs (17 males, age: 31.8 ± 8.1). The two groups had no significant differences in age (*p* = 0.8552), years of education (*p* = 0.5691) and gender (*p* = 0.8638). Compared with the LDH-pre patients, the LDH-pos patients had significantly lower VAS scores (*p* < 0.0001) and C-SFODI scores (*p* < 0.0001) (Details are shown in Table 1).

**TABLE 1:** Demographic characteristics of the LDH patients and HC groups.

	LDH	HCs	*P*-value
Participants	27	28	-
Gender (male\female)	(17\10)	(17\11)	0.8638^a^
Age (year)	32.2 ± 9.5	31.8 ± 8.1	0.8552^b^
Years of education (year)	16.04 ± 1.93	16.36 ± 2.20	0.5691^b^
VAS scores (LDH-pre\pos)	(5.6 ± 2.1\1.7 ± 1.1)	-	<0.0001^c^
C-SFODI scores (LDH-pre\pos)	(26.9 ± 8.1\18.7 ± 5.4)	-	<0.0001^c^

^a^ *χ*2 test; ^b^ Two sample *t*-test; ^c^ Paired t-test between LDH patients before and after Tuina treatment. LDH, lumbar disc herniation; HCs, healthy controls; VAS, visual analogue scale; C-SFODI: the Chinese Short Form Oswestry Disability Index Questionnaire.

### Differences in regional homogeneity between LDH-pre and HCs

**FIGURE 2:**
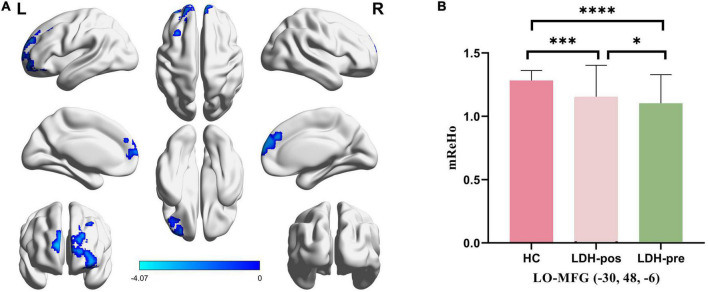
**(A)** Significantly different regions in ReHo between LDH-pre patients and HCs. **(B)** The bar graphs represent the mean ReHo extracted from abnormal clusters. The LDH patients and HCs were compared using two-sample *t*-tests, and LDH patients before and after Tuina were compared by paired *t*-tests. * indicated significance level at *p* < 0.05 (two-tailed), ^***^ indicated significance level at *p* < 0.001 (two -tailed), ^*⁣*⁣**^ indicated significance level at *p* < 0.0001 (two -tailed). HC, healthy controls; LDH-pos, LDH patients after Tuina; LDH-pre, LDH patients before Tuina; LO-MFG, left orbital part middle frontal gyrus.

The result of ReHo analysis showed that LDH-pre patients had decreased ReHo values in the left orbital part middle frontal gyrus (LO-MFG, Montreal Neurological Institute (MNI) coordinate: −30 48 −6) (Table 2 and Figure 2).

**TABLE 2:** Significantly different regions in ReHo between LDH-pre patients and HCs.

Brain area	Voxel size	Peak (MNI,x,y,z)	Peak *T* value	*P* value
LO-MFG	727	−30 48 −6	−4.0725	0.0002

ReHo, regional homogeneity; LDH-pre, lumbar disc herniation patients before Tuina treatment; HCs, healthy controls; LO-MFG, the left orbital part middle frontal gyrus; MNI, Montreal Neurological Institute.

### Differences in static FC analysis between LDH-pre and HCs

LO-MFG was selected as a seed to compare the FC between LDH-pre and HCs. We didn’t find any significant difference regions between LDH-pre patients and HCs after GRF multiple comparisons correction.

### Differences in variability of dFC analysis between LDH-pre and HCs

**FIGURE 3:**
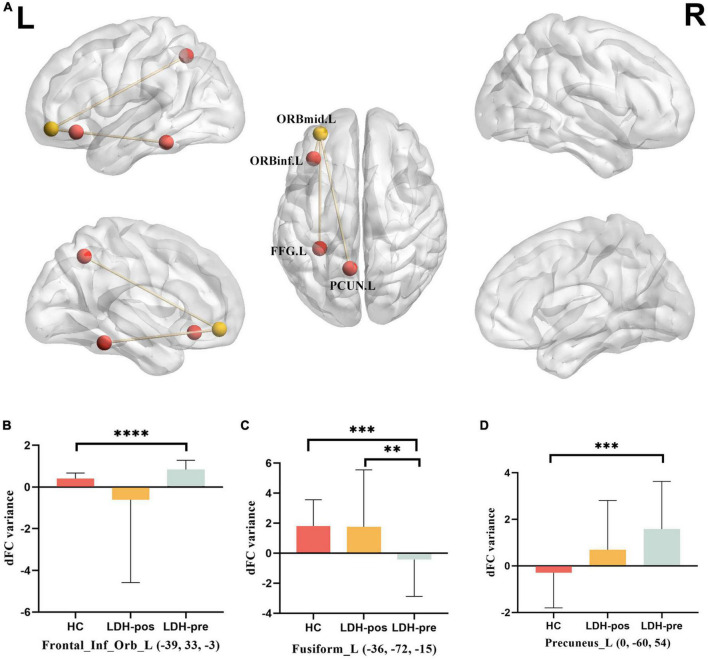
**(A)** Brain regions showing group differences between LDH_pre and HCs in dynamic FC variance with the LO-MFG as seed. **(B)** DFC variance located in Frontal_Inf_Orb_L (compare each group with every other group). **(C)** DFC variance located in Frontal_Inf_Orb_L (compare each group with every other group). **(D)** DFC variance located in Precuneus_L (compare each group with every other group). The LDH patients and HCs were compared using two-sample *t*-tests, and LDH patients before and after Tuina were compared by paired *t*-tests. ^**^ indicated significance level at *p* < 0.01 (two -tailed), ^***^ indicated significance level at *p* < 0.001 (two -tailed), ^*⁣*⁣**^ indicated significance level at *p* < 0.0001 (two -tailed). HC, healthy controls; LDH-pos, LDH patients after Tuina; LDH-pre, LDH patients before Tuina; LO-MFG, left orbital part middle frontal gyrus. Frontal_Inf_Orb_L, left orbital inferior frontal gyrus; Fusiform_L, left Fusiform; Precuneus_L, left Precuneus.

LO-MFG was selected as a seed to compare the dFC variance between LDH-pre and HCs. Two-sample *t*-test revealed LDH-pre patients shown decreased dFC variance in left Fusiform, increased dFC variance located in left orbital inferior frontal gyrus and left precuneus, compared to the HCs (Table 3 and Figure 3).

**TABLE 3:** Significantly different regions in dFC variance between LDH-pre patients and HCs.

Seed region	Connected regions	Voxel size	Peak (MNI,x,y,z)	Peak *T*-value	*P*- value
LO-MFG (−30 48 −6)	Fusiform_L	10	−36, −72, −15	-2.5631	0.0132
	Frontal_Inf_Orb_L	37	−39, 33, −3	3.3115	0.0017
	Precuneus_L	10	0, −60, 54	2.833	0.0065

LDH-pre, lumbar disc herniation patients before Tuina treatment; HCs, healthy controls; LO-MFG, left orbital part middle frontal gyrus; Fusiform_L, left Fusiform; Frontal_Inf_Orb_L, left orbital inferior frontal gyrus; Precuneus_L, left Precuneus; MNI, Montreal Neurological Institute.

### Assessment of Tuina intervention

We extracted mReHo values within LO-MFG mask from LDH-pre, LDH-pos and HCs separately. LDH-pos patients had increased ReHo compared with LDH-pre patients (*p* = 0.029908). Meanwhile, both the LDH-pre and LDH-pos patients showed significantly decreased ReHo compared with the HCs (*p* < 0.0001 and *p* < 0.001) (Table 4 and Figure 2).

**TABLE 4:** Group differences in mean ReHo values.

Coordinates	Brain region	Contrast	*P*-value	*T*-value
−30 48 −6	LO-MFG	LDH-pre vs. HCs	<0.0001	−7.56100 2.297216 −3.53306
		LDH-pos vs. LDH-pre	0.0299	
		LDH-pos vs. HCs	0.0009	

LO-MFG, the left orbital part middle frontal gyrus; LDH-pre, lumbar disc herniation patients before Tuina treatment; HCs, healthy controls; LDH-pos, lumbar disc herniation patients after Tuina treatment.

We extracted mean dFC variance values within left Fusiform, left orbital inferior frontal gyrus and left precuneus mask from LDH-pre, LDH-pos and HCs separately. In left Fusiform, LDH-pos patients had increased dFC variance compared with LDH-pre patients (*p* = 0.009), no significant difference compared with HCs (*p* = 0.9796). Meanwhile, LDH-pre showed significantly decreased dFC variance compared with the HCs (*p* = 0.0004). In left orbital inferior frontal gyrus, LDH-pre patients had increased dFC variance compared with HCs (*p* < 0.0001), no significant difference compared with LDH-pos and HCs (*p* = 0.0827 and *p* = 0.1891). In left precuneus, LDH-pre patients had increased dFC variance compared with HCs (*p* = 0.0002), no significant difference compared with LDH-pos and HCs (*p* = 0.1279 and *p* = 0.0529) (Table 5 and Figure 3).

**TABLE 5:** Group differences in mean dFC variance values.

Seed region	Connected regions	Coordinates	Contrast	*P*-value	*T*-value
LO-MFG (-30 48 -6)	Fusiform_L	−36, −72, −15	LDH-pre vs. HCs	0.0004	-3.8158
			LDH-pos vs. LDH-pre	0.0090	2.8234
			LDH-pos vs. HCs	0.9796	0.0257
	Frontal_Inf_ Orb_L	−39, 33, −-3	LDH-pre vs. HCs	<0.0001	4.6484
			LDH-pos vs. LDH-pre	0.0827	-1.8049
			LDH-pos vs. HCs	0.1891	-1.3303
	Precuneus_L	0, −60, 54	LDH-pre vs. HCs	0.0002	3.9325
			LDH-pos vs. LDH-pre	0.1279	-1.5722
			LDH-pos vs. HCs	0.0529	1.9802

LDH-pre, lumbar disc herniation patients before Tuina treatment; LDH-pos, lumbar disc herniation patients after Tuina treatment; HCs, healthy controls; LO-MFG, left orbital part middle frontal gyrus; Fusiform_L, left Fusiform; Frontal_Inf_Orb_L, left orbital inferior frontal gyrus; Precuneus_L, left Precuneus.

## Discussion

### Main results of this study

In this study, rs-fMRI was used in combination with ReHo and seed-based FC analysis to examine the coherence of local neural activity and whole brain connectivity in LDH patients. Next, using this information as a basis, we evaluated the effective of Tuina intervention. Compared with HCs, patients with LDH had reduced ReHo in LO-MFG and anomalous variance of dFC between LO-MFG and three brain regions, including the central brain region of the default mode network (the left precuneus), the medial prefrontal cortex (mPFC) (the left orbital inferior frontal gyrus), and the left fusiform cortex. After Tuina treatment, the coherence of LO-MFG neural activity of LDH patients increased and their dFC variability was also improved.

### LDH patients may have default mode network dysfunction

We finded that he ReHo in the LO-MFG, located in the mPFC, was lower in LDH patients, agreeing with the result from Zhou et al. (Zhou et al., 2019). The reduced ReHo in LO-MFG could indicate that this gene is involved in the pathology of LDH and that its ability to regulate emotion is deteriorating (Zhao et al., 2019). The decreased activity of the LO-MFG may result in a weakened impact on mood regulation, an increase in anxiety and depression, and a reduced ability to use emotion adjustment techniques to alter the amygdala’s response to harmful stimuli (Rolls, 2019).

The mPFC form a hub (or core) node of the DMN, and disrupted connectivity of the DMN in chronic low back pain (Huang et al., 2019; Ng et al., 2021). We selected the LO-MFG seed to calculate static and dynamic functional connectivity. The dFC variance of left orbital inferior frontal gyrus and left precuneus were larger in LDH patients compared to HC. The left fusiform dFC variance was lower in LDH patients than in HCs. The fusiform, a portion of the temporal and occipital lobes, is known to be engaged in several neuronal pathways that are involved in recognition (Jonas et al., 2015; Beelen et al., 2022), memory (Liu et al., 2021; Yan et al., 2022), and emotion (Liu et al., 2021; Yan et al., 2022), despite the fact that its role is not entirely understood. The orbital inferior frontal gyrus and the precuneus are both a member of the standard mode network. When the brain is at rest, the standard network serves as the main hub of functional connections across brain regions, monitoring both internal and external environments, episodic memory, and ongoing cognitive and emotional activity (Brewer et al., 2011). Numerous studies have shown that individuals with chronic low back pain have aberrant brain areas and functional connections associated to miswiring (Zhang S. et al., 2014; Ng et al., 2021). Additionally, Zhang and colleagues demonstrated that patients with cLBP had increased ALFF in the hippocampal/parahippocampal gyrus but a decrease in ALFF values in the remaining gyrus DMN regions when their spontaneous low back pain increased after the pain amelioration maneuver (Zhang et al., 2019). In comparison to healthy controls, individuals with discogenic leg pain had decreased ALFF values in DMN areas, according to Zhou and colleagues (Zhou et al., 2018). This means that a DMN problem may also be the root of the cognitive and behavioral issues identified in LDH.

### The disordered default mode network can be functionally reversed by Tuina

Tuina has a strong analgesic response when used to treat chronic LDH (Kong et al., 2012). Previous research on the mechanism of analgesia has mainly concentrated on the biomechanical effect, which is hypothesized to be the main therapeutic mechanism. For example, Du et al. investigated the effect of lumbar manipulations with finite element technique, showing the relative displacement between the intervertebral disc and adjacent nerve roots and the internal stress change of the lumbar intervertebral disc (Du et al., 2016). But for pain relief, the central nervous system must be mediated (Ng et al., 2021). The default mode network is part of the triple network model which explains the whole range of pain related co-morbidities (De Ridder et al., 2022). To clear the effect of Tuina on the default mode network in LDH patients is of great importance to understand the central mechanism of Tuina for pain relief.

In the past, the resting magnetic resonance studies on the effect of Tuina mainly focused on changes in spontaneous brain activity or functional connectivity, but not on whether Tuina can restore abnormal brain activity or functional connectivity to a normal level. Actually, to prove that Tuina can reverse the brain function damaged by disease, is of great significance to reveal the remodeling of nervous functions mechanism of Tuina effect. We finded that after effective Tuina intervention, the ReHo value of the LO-MFG decrease in LDH patients increased. The dFC variability of the default mode network anomalies in LDH patients also improved at the same time. Our results lend credence to the notion that the DMN activity is the neural correlate of LDH and that the control of dFC in important DMN brain areas may help to explain the analgesic effect of Tuina and the brain response of LDH patients to pain management after Tuina.

### Limitations

Our study has several drawbacks as well. First, because we applied tight inclusion and exclusion criteria, our sample size was small. Future studies should examine local synchronous activity anomalies in LDH patients using a larger sample size. Second, since there was no intervention in HCs, we assumed that the fMRI data has no change within two weeks of the study period, but this may not be the case. In future research, a group of HCs receiving manipulation of intervention can be added. Last, following the sixth TMS, we did not obtain any follow-up information from patients with LDH. Future studies might involve lengthier follow-ups to obtain data on the duration of pain relief after Tuina.

## Conclusion

In comparison to HCs, LDH patients have decreased regional homogeneity of major brain regions of the default mode network and disorder functional connectivity. Our research indicated that Tuina can reshape the function of the default mode network (DMN) in LDH patients, which may contribute to the analgesic effect of Tuina in LDH patients.

## Data availability statement

The raw data supporting the conclusions of this article will be made available by the authors, without undue reservation.

## Ethics statement

The studies involving human participants were reviewed and approved by the Medical Research Ethics Committee of the First Clinical Medical College of the Zhejiang Chinese Medical University. The patients/participants provided their written informed consent to participate in this study. Written informed consent was obtained from the individual(s) for the publication of any identifiable images or data included in this article.

## Author contributions

H-GD and ZJ designed the study. YW collected the data. X-MC analyzed the data. X-MC and YW wrote the first version. SC, CL, WW, and NK revised the manuscript. X-MC, YW, XJ, D-YL, QH, J-EC, X-LZ, JL, M-SX, ZJ, and H-GD added their comments. All authors approved the manuscript.

## Footnotes

1
http://restfmri.net/forum/DynamicBC
